# CART for ascites accumulation and dialysis difficulty after hepatic cyst fenestration and bilateral nephrectomy in ADPKD: a case report

**DOI:** 10.1007/s13730-026-01113-6

**Published:** 2026-04-07

**Authors:** Saori Sawada, Sho Sugahara, Anna Sumii, Tomonori Sakae, Aki Yamada, Yoshimi Imamura-Uehara, Nahomi Ishimoto, Shogo Kuwagata, Kosuke Yamahara, Mako Yasuda-Yamahara, Yuki Tanaka-Sasaki, Masami Chin-Kanasaki, Shinji Kume

**Affiliations:** Division of Diabetes, Endocrinology and Nephrology, Department of Medicine, Shiga University of Medical Science, Seta Tsukinowa-Cho, Otsu, Shiga 520-2192 Japan

**Keywords:** Autosomal dominant polycystic kidney disease, Ascites accumulation, Cell-free and concentrated ascites reinfusion therapy, Hepatic cyst fenestration

## Abstract

Hepatomegaly with hepatic cysts constitutes a common extrarenal manifestation of autosomal dominant polycystic kidney disease (ADPKD), often resulting in mass effects and occasionally necessitating cyst fenestration to alleviate abdominal distension. Nonetheless, persistent cyst fluid leakage as a postoperative complication frequently leads to refractory ascites, for which treatment options are limited. In this report, we present a 62-year-old female patient with ADPKD undergoing maintenance hemodialysis. She received bilateral nephrectomy and hepatic cyst fenestration to mitigate abdominal distension. Postoperatively, she developed ascites accumulation and recurrent intradialytic hypotension, impairing effective ultrafiltration. The ascites proved unresponsive to conservative management, and analysis indicated hepatic cyst fluid leakage, with elevated CA19-9 levels in the ascitic fluid. Repeated ultrafiltration and abdominal paracentesis failed to produce sustained benefits. Subsequently, she underwent two sessions of cell-free and concentrated ascites reinfusion therapy (CART), which successfully reduced her ascites. Over the subsequent four months, her serum albumin levels increased, intradialytic hypotension resolved, and the ascites subsided without recurrence, thus permitting the repair of an inguinal hernia identified at referral. This case exemplifies that CART constitutes an effective therapeutic modality for managing ascites accumulation resulting from hepatic cyst leakage following fenestration in patients with ADPKD.

## Background

Autosomal dominant polycystic kidney disease (ADPKD) is the most prevalent inherited renal disorder globally and represents a principal cause of end-stage renal disease (ESRD). In Japan, its prevalence is estimated at 1 in 4000 individuals [1], accounting for 2–3% of cases requiring dialysis initiation. While the progression to ESRD constitutes a major clinical concern in ADPKD, hepatomegaly resulting from hepatic cysts, coupled with considerably enlarged kidneys, can also lead to symptoms such as abdominal distension, early satiety, shortness of breath, or complications arising from compression of the inferior vena cava or portal vein [2, 3]. These conditions markedly diminish the quality of life among patients with ADPKD, thereby rendering the management of mass effects, including abdominal symptoms, a significant clinical challenge.

To mitigate these abdominal symptoms, surgical interventions such as nephrectomy or hepatic cyst fenestration are occasionally contemplated [4–6]. However, each procedure entails potential risks. For example, bilateral nephrectomy may ablate the renin–angiotensin–aldosterone (RAAS) system, potentially disrupting fluid homeostasis [7, 8]. Hepatic cyst fenestration has been associated with postoperative complications including persistent cyst fluid leakage, bile leakage, and refractory ascites [9]. Significantly, no definitive treatment exists for refractory ascites resulting from postoperative cyst fluid leakage subsequent to hepatic cyst fenestration.

The clinical utility of cell-free and concentrated ascites reinfusion therapy (CART) has recently been evidenced in the management of refractory and malignant ascites [10]. CART is a minimally invasive and repeatable procedure that reinfuses concentrated ascitic fluid, thereby aiding in the preservation of valuable serum proteins and potentially improving quality of life [10, 11]. Nonetheless, to date, there have been no reports demonstrating the efficacy of CART for refractory ascites subsequent to hepatic cyst fenestration in patients with ADPKD. Herein, we present a case of ADPKD in which CART effectively managed refractory ascites and dialysis intolerance due to intradialytic hypotension following concomitant bilateral nephrectomy and hepatic cyst fenestration.

## Case presentation

A 62-year-old woman with a history of ADPKD developed ESRD and has been undergoing maintenance hemodialysis since year X-5. Six months prior to admission to the nephrology department, she was referred from her maintenance dialysis hospital to the Departments of Gastrointestinal Surgery and Urology because of severe abdominal distension and early satiety caused by massively enlarged polycystic kidneys and multiple hepatic cysts (Fig. 2). Based on the assessment by both departments, she underwent simultaneous bilateral nephrectomy and hepatic cyst fenestration at our institution. During the procedure, two dominant hepatic cysts (measuring 9.6 cm and 9.3 cm in maximal diameter) were fenestrated, although numerous smaller cysts were also present. Following the procedure, she returned to a local dialysis center for maintenance hemodialysis. Although her symptoms temporarily improved postoperatively, she subsequently experienced abdominal distension accompanied by ascites, as well as generalized fatigue, anorexia, tachycardia, and orthostatic hypotension. After bilateral nephrectomy, her blood pressure was approximately 90–100/60–70 mmHg, and she developed severe intradialytic hypotension resistant to droxidopa and amezinium, making adequate ultrafiltration difficult. After CART and improvement of serum albumin levels, her blood pressure gradually stabilized and intradialytic hypotension was resolved. She was consequently referred to our nephrology department in year X and was admitted on the same day for further assessment.

Upon admission (Day 1), her height was 167.1 cm, and her weight was 57.6 kg, with a body mass index of 20.6 kg/m^2^. She was alert and oriented. Physical examination revealed mild anemia, a markedly distended and firm abdomen, and bilateral leg edema. Her blood pressure was low, even outside of dialysis (97/66 mmHg). Laboratory findings indicated mild anemia and normal leukocyte and platelet counts. Renal function was severely compromised, as evidenced by elevated blood urea nitrogen and serum creatinine levels. Conversely, liver function remained unaffected, demonstrated by normal levels of aspartate aminotransferase, alanine aminotransferase, and total bilirubin. Electrolyte levels, including sodium, potassium, chloride, calcium, and phosphorus, were within normal limits. C-reactive protein was slightly elevated. The patient exhibited hypoalbuminemia, and the CA19-9 level was markedly elevated. At the time of admission, the patient’s serum γ-GTP level was elevated (288 U/L), whereas total bilirubin and AST/ALT levels were within normal limits. The ascitic fluid appeared clear yellow without bile staining.

Diagnostic paracentesis was performed for the differential diagnosis of ascites. The ascitic fluid appeared clear yellow with exudative features (TP 4.3 g/dL, SAAG 0.8 g/dL), and CA19-9 was significantly elevated (2517 U/mL), suggesting hepatic cyst leakage (Table 1). No signs of bile leakage or malignancy were detected. Since the cyst wall had been resected during fenestration, closure was not possible; thus, surgical repair was not performed.

**Table 1:** Ascitic fluid analysis

Appearance: Clear yellow	
Cytology: No malignant cells detected	
Bacterial culture: Negative	
Specific gravity: 1.018	
*Biochemistry*	
TP	4.3 g/dL
Alb	2.2 g/dL
LD	108 U/L
T-Bil	0.5 mg/dL
T-Cho	103 mg/dL
TG	27 mg/dL
AMY	59 U/L
CA 19-9	2517 U/mL
CEA	3.5 ng/mL
ADA	14.4 U/L
SAAG	0.8 g/dL
Cell count	117 /μL
Neutrophils	0.5%
Lymphocytes	10.5%
Monocytes	7.5%
Eosinophils	0.0%
Basophils	1.0%
Macrophages	80.5%

TP, total protein; Alb, albumin; LD, lactate dehydrogenase; T-Bil, total bilirubin; T-Cho, total cholesterol; TG, triglyceride; AMY, amylase; CA 19-9, carbohydrate antigen 19-9; CEA, carcinoembryonic antigen; ADA, adenosine deaminase; SAAG, serum–ascites albumin gradient

Considering these findings, the indications for therapeutic paracentesis or continuous drainage were evaluated; however, since the ascites was thought to result from persistent leakage after hepatic cyst fenestration, such procedures were deemed to carry a high risk of significant albumin loss. Therefore, initial management focused on gradual ultrafiltration during dialysis to achieve a fluid balance compatible with daily activities and to improve appetite. With ongoing predialysis droxidopa to prevent intradialytic hypotension, we attempted stepwise reductions in dry weight (DW) from 57.0 to 55.7 kg. However, abdominal distension and subjective symptoms continued, and intradialytic hypotension worsened. As a result, the DW was adjusted back to 56.5 kg. Given her post-bilateral nephrectomy, the lack of the renin–angiotensin-aldosterone (RAAS) system combined with hypoalbuminemia was thought to contribute to intradialytic hypotension; plasma renin activity and serum aldosterone levels were both undetectable (Table 2).

**Table 2:** Laboratory findings on admission

*Complete blood count (CBC)*		AST	21 U/L
WBC	3900/μL	ALT	14 U/L
RBC	319 × 10^4^/μL	ALP	188 IU/L
Hb	10.0 g/dL	LD	157 U/L
Ht	32.6%	T-Bil	0.5 mg/dL
Plt	350 × 10^3^/μL	BUN	37.6 mg/dL
*Blood chemistry*		Cr	6.57 mg/dL
Na	139 mmol/L	BUN	37.6 mg/dL
K	4.2 mmol/L	Cr	6.57 mg/dL
Cl	104 mmol/L	eGFR	5.6 mL/min/1.73m^2^
Ca	8.3 mg/dL	CRP	1.88 mg/dL
P	4.5 mg/dL	Plasma renin activity	< 0.2 ng/ml/h
TP	5.4 g/dL	Aldosterone	< 4.0 pg/mL
Alb	2.3 g/dL	CA 19-9	924 U/mL

WBC, white blood cell count; RBC, red blood cell count; Hb, hemoglobin; Ht, hematocrit; Plt, platelet count; AST, aspartate aminotransferase; ALT, alanine aminotransferase; ALP, alkaline phosphatase; LD, lactate dehydrogenase; T-Bil, total bilirubin; BUN, blood urea nitrogen; Cr, creatinine; Na, sodium; K, potassium; Cl, chloride; eGFR, estimated glomerular filtration rate; Ca, calcium; P, phosphorus; CRP, C-reactive protein; TP, total protein; Alb, albumin; CA 19-9, carbohydrate antigen 19-9

During the first hospitalization (Day 1–27), the restriction of dietary salt and fluids resulted in a temporary improvement in interdialytic weight gain, thereby alleviating dialysis intolerance and enabling her discharge. However, after returning to maintenance dialysis at the outpatient dialysis facility, the ascites and abdominal distension persisted, leading to a second hospitalization (Day 215–218). During this second admission, we decided to perform a CART. The first CART session processed 2,080 mL with 330 mL reinfused and was completed without complications. Since her symptoms initially improved, maintenance dialysis was resumed; however, with reaccumulation of ascites, the patient was hospitalized for a third time (Day 308–311) and underwent a second CART session. The second session processed 3,000 mL with 510 mL reinfused.

**Fig. 1:**
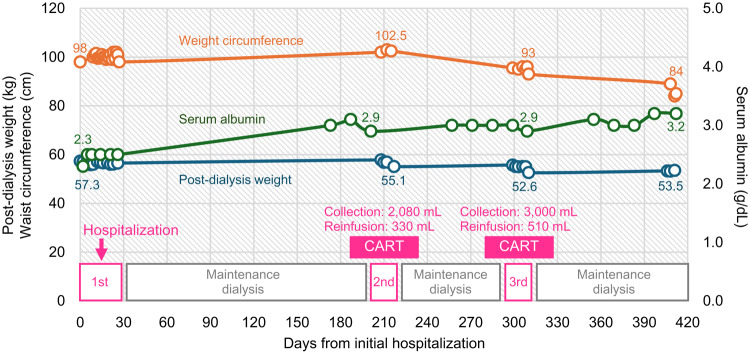
Clinical course of the present case after the first admission. The present case experienced three hospitalizations. Based on information from the maintenance dialysis facility and data obtained at our hospital, the changes in abdominal circumference, post-dialysis body weight, and serum albumin levels are shown. Cell-free and concentrated ascites reinfusion therapy (CART) was performed during the second and third hospitalizations

**Fig. 2:**
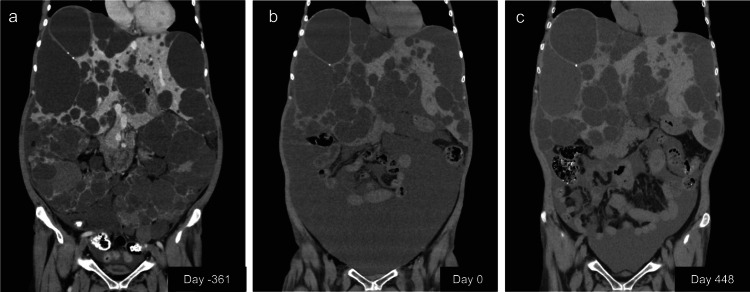
Coronal abdominal computed tomography (CT) findings. **a** Preoperative (day -361) CT image demonstrating markedly enlarged polycystic kidneys and multiple hepatic cysts, resulting in severe abdominal distension. **b** CT image obtained at admission (day 0), showing massive ascites. **c** CT image obtained on day 448, 140 days after the second CART session, showing a reduction in ascites

Following two CART sessions, the abdominal circumference decreased from 102.5 cm to 93 cm (Fig. 1). Notably, by Day 410, the patient’s abdominal circumference had reduced to 84 cm without any medical intervention (Fig. 1). Moreover, the substantial ascites observed on computed tomography during the initial hospitalization remained markedly diminished even after the second CART session (Fig. 2). Ultimately, all modalities of ascites management, including CART, ceased to be necessary. During this period, the serum albumin level increased (Fig. 1), intradialytic hypotension was resolved, and stable maintenance dialysis was achieved. Importantly, no albumin infusion or other albumin-augmenting therapy was administered during this period. Furthermore, surgical repair of the inguinal hernia, identified at the time of initial hospitalization, was carried out.

## Discussion and conclusions

We present a case of a patient who underwent simultaneous bilateral nephrectomy and hepatic cyst fenestration to relieve abdominal symptoms. This procedure not only resulted in the loss of the RAAS-mediated mechanisms responsible for fluid and blood pressure homeostasis but also led to refractory ascites caused by persistent cyst fluid leakage, significantly impairing the patient's quality of life. Although conservative management proved ineffective, two sessions of CART successfully achieved a sustained reduction in ascites, enabling a transition to stable maintenance dialysis. Nephrectomy and fenestration of hepatic cysts each have established indications for improving QOL by alleviating abdominal distension in ADPKD [5, 12]; however, performing both procedures, particularly concurrently, may result in refractory ascites, as observed in this case, which can further impair QOL. The potential disadvantages associated with combined surgical interventions should therefore be carefully considered when choosing the best treatment approach.

In addition, both bilateral nephrectomy and hepatic cyst fenestration are invasive procedures associated with a non-negligible risk of perioperative morbidity in patients with ADPKD. Reported complications include bleeding, infection, persistent ascites, and bile leakage or other biliary complications [9, 13]. Therefore, careful risk–benefit assessment and appropriate patient selection are essential when considering these procedures, particularly in individuals who have already progressed to ESRD [14]. Less invasive strategies, including medical management and, in selected cases, volume-reducing interventions such as renal transcatheter arterial embolization [15], may also be considered as alternative options. Our case highlights the importance of multidisciplinary evaluation and shared decision-making when surgical intervention is contemplated for symptom relief in patients with ADPKD.

In this context, renal transcatheter arterial embolization (TAE) is sometimes considered as a less invasive option to reduce kidney volume in ADPKD. However, in this patient, the surgical team selected simultaneous bilateral nephrectomy and hepatic cyst fenestration to achieve rapid and reliable decompression because she had already progressed to end-stage renal disease and had massive enlargement of both kidneys and liver due to cystic disease.

CART is a widely utilized therapeutic modality for refractory ascites, especially in cases related to liver cirrhosis and malignancy [10, 16]. The procedure involves the extracorporeal filtration and concentration of ascitic fluid, followed by the reinfusion of autologous proteins, thereby mitigating protein loss and hypoalbuminemia commonly associated with recurrent paracentesis [17]. Although CART necessitates several hours per session, it can be seamlessly incorporated into dialysis sessions for patients undergoing maintenance dialysis. To date, however, there have been no reports confirming the efficacy of CART in managing ascites subsequent to hepatic cyst fenestration in patients with ADPKD.

In our patient, markedly elevated CA19-9 in the ascitic fluid suggested hepatic cyst leakage as the primary cause of ascites [18, 19], and the site of leakage could not be identified surgically. Additionally, she experienced hypoalbuminemia (2.3 g/dL) and unstable blood pressure resulting from the loss of RAAS activity following bilateral nephrectomy, rendering intradialytic hypotension a frequent and debilitating complication and suggesting a multifactorial basis for the development and persistence of ascites. In anephric patients, persistent hypotension may result not only from the complete loss of RAAS activity but also from interruption of renal sympathetic afferent signaling, which is known to influence systemic hemodynamics.

**Fig. 3:**
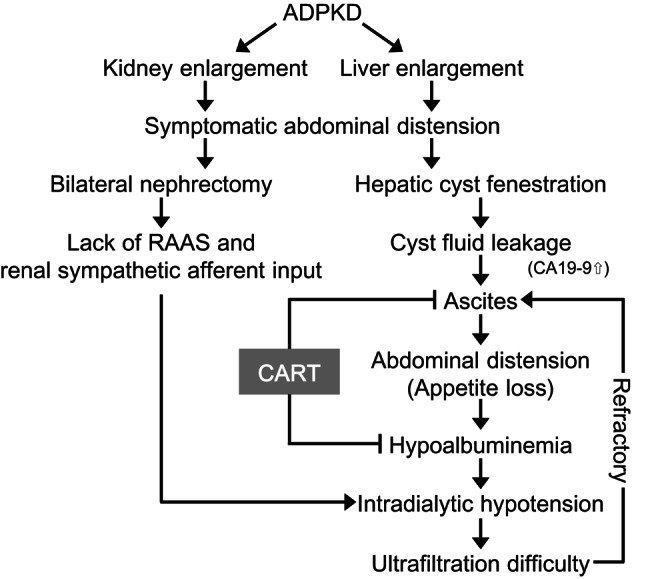
Schematic representation of the vicious cycle of refractory ascites and its resolution by CART. In a patient with ADPKD, bilateral nephrectomy and hepatic cyst fenestration resulted in persistent cyst fluid leakage and ascites with elevated CA19-9 levels, leading to loss of appetite. Ascites management by ultrafiltration was hindered by hypoalbuminemia and impaired hemodynamic regulation due to loss of the renin–angiotensin–aldosterone system (RAAS), causing intradialytic hypotension and refractory ascites in a vicious cycle. Cell-free and concentrated ascites reinfusion therapy (CART) reduced ascites volume and improved hypoalbuminemia, thereby breaking this cycle

Although simple abdominal paracentesis is a standard option for refractory ascites, we avoided this approach in the present case because of the high risk of progressive albumin loss. Given these circumstances, CART was deemed the most suitable intervention, as it facilitated both the safe reduction of ascitic fluid volume and the preservation of oncotic pressure. After two CART sessions, her abdominal distension significantly improved, serum albumin increased to 3.2 g/dL, and intradialytic hypotension was resolved. The rise in serum albumin following CART in our patient was achieved without exogenous albumin supplementation, suggesting that preservation and reinfusion of autologous protein contributed to restoring oncotic pressure. Although regular CART was initially anticipated, the unexpected improvement in albumin levels and hemodynamic stability permitted sustained management with only two sessions, and no further procedures have been required for more than 10 months after the second CART session. This outcome suggests that CART not only alleviated ascites but also helped interrupt the vicious cycle of hypoalbuminemia and ultrafiltration intolerance, thereby contributing to long-term stability (Fig. 3). If ascites were to recur, CART would again be considered because it allows protein-sparing fluid removal in hemodialysis patients, while standard measures such as dietary salt restriction and appropriate fluid management remain important for preventing recurrent fluid accumulation.

In conclusion, this is the first report to demonstrate the effectiveness of CART in managing ascites accumulation after hepatic cyst fenestration and bilateral nephrectomy in a patient with ADPKD, highlighting the potential usefulness of CART for treating refractory ascites in such cases.

## Data Availability

No datasets were generated or analyzed during the current study.

## Abbreviations

ADPKD: Autosomal dominant polycystic kidney disease
Alb: Albumin
CA19-9: Carbohydrate antigen 19-9
CART: Cell-free and concentrated ascites reinfusion therapy
CT: Computed tomography
DW: Dry weight
ESRD: End-stage renal disease
PRA: Plasma renin activity
RAAS: Renin–angiotensin–aldosterone system
SAAG: Serum-ascites albumin gradient
